# Not in the drug, not in the brain: Causality in psychedelic experiences from an enactive perspective

**DOI:** 10.3389/fpsyg.2023.1100058

**Published:** 2023-04-03

**Authors:** Daniel Meling, Milan Scheidegger

**Affiliations:** ^1^Department of Psychiatry, Psychotherapy and Psychosomatics, Psychiatric University Hospital Zurich, University of Zurich, Zurich, Switzerland; ^2^Department of Psychosomatic Medicine and Psychotherapy, Faculty of Medicine, Medical Center – University of Freiburg, Freiburg, Germany; ^3^Neuroscience Center Zurich (ZNZ), University of Zurich, Zurich, Switzerland

**Keywords:** psychedelics, neuroscience, phenomenology, drug, enaction, causality, psychedelic therapy, biopsychosocial model

## Abstract

Psychedelics are psychoactive substances that receive renewed interest from science and society. Increasing empirical evidence shows that the effects of psychedelics are associated with alterations in biochemical processes, brain activity, and lived experience. Still, how these different levels relate remains subject to debate. The current literature presents two influential views on the relationship between the psychedelic molecule, neural events, and experience: The integration view and the pluralistic view. The main aim of this article is to contribute a promising complementary view by re-evaluating the psychedelic molecule-brain-experience relationship from an enactive perspective. We approach this aim *via* the following main research questions: (1) What is the causal relationship between the psychedelic drug and brain activity? (2) What is the causal relationship between brain activity and the psychedelic experience? In exploring the first research question, we apply the concept of *autonomy* to the *psychedelic molecule-brain relationship.* In exploring the second research question, we apply the concept of *dynamic co-emergence* to the *psychedelic brain-experience relationship*. Addressing these two research questions from an enactive position offers a perspective that emphasizes interdependence and circular causality on multiple levels. This enactive perspective not only supports the pluralistic view but enriches it through a principled account of how multi-layered processes come to interact. This renders the enactive view a promising contribution to questions around causality in the therapeutic effects of psychedelics with important implications for psychedelic therapy and psychedelic research.

## 1. Introduction

The recent resurgence of psychedelic research shows promising evidence of the clinical benefits of psychedelics. This is likely to increase the use of psychedelic treatments in psychiatry and society at large. Motivated by the clinical potential of psychedelics, basic research into pharmacological mechanisms, neuroscientific underpinnings, and into the phenomenological features of psychedelics and their therapeutic outcomes has become a research priority. While increasing empirical evidence shows that the effects of psychedelics are associated with their partial agonism for the serotonin 5-HT_2A_ receptors, with substantial alterations in brain activity, and with certain aspects of the subjective experience, there are two different views in the current literature on how these various processes on different levels relate: The first view, the *integration* view, concludes that the different levels converge on a similar linear causal cascade: The psychedelic molecule creates changes in brain activity and the brain creates the psychedelic experience. The second view, the pluralistic view, however, holds that psychedelic experiences cannot be reduced to a single causal pathway and argues instead for the interplay of multiple causal pathways. In this article, we aim to contribute to this debate through re-evaluating the psychedelic molecule-brain-experience relationship from a promising third perspective: the enactive approach. Enactive cognitive science provides a framework of interrelated ideas that emphasize the lack of ultimate foundations on various levels: Following the thread of interdependence, the enactive approach questions the causally linear and reductionist notions of various seemingly dualistic relationships, including those between parts and whole, self and world, and subject and object.

### 1.1. Psychedelics and their relevance

Psychedelics are psychoactive substances that temporarily alter perception, emotions, cognition, and the sense of self (Griffiths et al., 2016; Carhart-Harris et al., 2016a,b; Muttoni et al., 2019).

After decades of dormancy, the number of publications on psychedelics has rapidly increased in recent years (Petranker et al., 2020; Hadar et al., 2022). A fast-growing body of clinical studies shows increasing evidence of promising therapeutic effects of psychedelics for various conditions (Goldberg et al., 2020; Romeo et al., 2020; Andersen et al., 2021; Lawrence et al., 2021; Hadar et al., 2022), including major depression (Carhart-Harris et al., 2016a, 2021; Davis et al., 2021), anxiety and depression in patients diagnosed with a life-threatening disease (Griffiths et al., 2016; Ross et al., 2016), and substance use disorders (Johnson et al., 2014, 2017; Bogenschutz et al., 2015, 2022). These results demonstrate the psychedelics’ potential for assisting psychotherapeutic processes (Garcia-Romeu and Richards, 2018; Reiff et al., 2020; Nayak and Johnson, 2021; Scheidegger, 2021b), rendering psychedelics a priority in psychiatric research and beyond, including pharmacological, neuroscientific, and phenomenological research.

### 1.2. State of psychedelic research: Pharmacology, neurobiology, and experience

Since these clinical results are promising, it is important to understand the specific conditions under which psychedelics’ therapeutic effects unfold. Pharmacological research is targeted toward the primary therapeutic mechanism of psychedelics on a pharmacological or biochemical level. Neuroscientific research is targeted toward the neurobiological mechanisms underlying psychedelic therapeutic experiences (Vollenweider and Preller, 2020; Vollenweider and Smallridge, 2022). In contrast, psychological and phenomenological research is targeted toward the subjective features of psychedelic experiences that predict therapeutic success (Roseman et al., 2018; Yaden and Griffiths, 2021). It is evident that pharmacological, neurobiological, and psychological research on psychedelics have gained renewed interest.

#### 1.2.1. Biochemical aspects of psychedelics

From a pharmacological perspective, classic serotonergic psychedelics can be defined with respect to their particular 5-HT_2A_ partial agonism (Nichols, 2016). This includes psilocybin, lysergic acid diethylamide (LSD), mescaline, and N,N-dimethyltryptamine (DMT). This particular 5-HT_2A_ partial agonism distinguishes classic serotonergic psychedelics from cannabinoids, and dissociatives such as ketamine, salvinorin A, and entactogens such as 3,4-methyl-enedioxymethamphetamine (MDMA), among other substances (Nichols, 2016). In addition to their 5-HT_2A_ partial agonism, classic psychedelics have been shown to increase levels of glutamate (Vollenweider and Kometer, 2010) and oxytocin (Holze et al., 2021a), to increase the production of brain-derived neurotrophic factor (BDNF) (De Almeida et al., 2019; Holze et al., 2020, 2021b), to promote neurogenesis (Ly et al., 2018), and to have anti-inflammatory effects (Nichols, 2016).

#### 1.2.2. Neurobiological aspects of psychedelics

From a neurobiological perspective, several theoretical frameworks have been proposed to account for psychedelic experiences. The three most influential are (1) the cortico-striato-thalamo-cortical (CSTC) model, (2) the claustro-cortical circuit (CCC) model, and (3) the relaxed beliefs under psychedelics (REBUS) model (van Elk and Yaden, 2022).

The CSCT model, an early account of the neurocognitive mechanisms underlying psychedelics, proposes the human brain usually exerts feedback loops between cortical areas and various thalamic nuclei, preventing an overload of information from outside and inside the brain (Vollenweider and Geyer, 2001). While the thalamic nuclei are proposed to work as a selective filter regulated by the prefrontal cortex, the CSCT model states that psychedelics release this inhibition of the prefrontal cortex over the thalamus, leading to an overload of information sent to other sensory brain regions (Vollenweider and Preller, 2020). In brief, the CSCT model assumes that psychedelics reduce the efficacy of thalamo-cortical filtering.

The CCC model, in contrast, is based on neuroimaging observations suggesting that psychedelics activate 5-HT_2A_ neurons in the claustrum which may cause a decoupling between prefrontal regions and the claustrum (Doss et al., 2022). The CCC model is supported by neuroimaging observations, suggesting that psilocybin resulted in significantly altered neural networks associated with cognitive control and with the functioning of the claustrum while subjective effects predicted changes in claustrum activity (Barrett et al., 2020). In brief, the CCC model suggests that psychedelics lead to an activation of the claustro-cortical circuit.

The REBUS model, on the other hand, is an influential account of psychedelic effects based on a synthesis of the entropic brain hypothesis and the free-energy principle (Carhart-Harris and Friston, 2019). Interestingly, the REBUS model not only provides an account on the neurobiological level but also a perspective on how to integrate it with multiple other levels, including the biochemical level and the experiential level. Therefore, the REBUS model could be understood as a twofold account: (1) as a neurobiological model and (2) as an integrative model on the relationship between various levels of analysis (see section Connecting the Various Levels of Psychedelic Effects). According to the REBUS model, psychedelics initiate a series of neurobiological changes on multiple levels (Carhart-Harris, 2019). On the molecular level, classic serotonergic psychedelics primarily affect serotonin 2A receptors (5-HT_2A_ receptors) (Carhart-Harris, 2019; Vollenweider and Preller, 2020). On the anatomical and functional level, this leads to increased neuroplasticity (Carhart-Harris, 2019; Banks et al., 2021), including changes in functional and directed connectivity between the thalamus and cortical areas (Müller et al., 2017; Preller et al., 2018, 2019; Vollenweider and Preller, 2020). On the dynamic level, increased entropy can be measured in certain aspects of brain function, indicating more unconstrained and less ordered neurodynamics (Carhart-Harris et al., 2014). On the systems level, network disintegration and desegregation are increased, i.e., global functional integration is increased (Petri et al., 2014; Palhano-Fontes et al., 2015; Tagliazucchi et al., 2016; Müller et al., 2018; Carhart-Harris, 2019), including increased synchrony of “sensory” brain regions and decreased integrity of “associative” brain regions, including the DMN and the frontoparietal control network (Carhart-Harris et al., 2012; Muthukumaraswamy et al., 2013; Palhano-Fontes et al., 2015; Kometer and Vollenweider, 2016; Carhart-Harris et al., 2016b; Müller et al., 2018; Preller et al., 2018, 2020; Lord et al., 2019; Vollenweider and Preller, 2020). Ultimately, the REBUS model uses a cascade of differentiating levels to describe how psychedelics lead to increased brain entropy. This reflects a loosening top-down weighting of priors and corresponds with increased liberation of bottom-up signaling which eventually culminates to a relaxation of high-level beliefs (Carhart-Harris, 2019; Carhart-Harris and Friston, 2019).

#### 1.2.3. Experiential aspects of psychedelics

On a more experiential level, psychedelics are associated with transient but significant alterations in perception, cognition, emotion, and the sense of self (Griffiths et al., 2016; Carhart-Harris et al., 2016a,b; Muttoni et al., 2019).

While there is a large variety of psychedelics-related subjective effects, recent research has mainly focused on a subset of psychedelic experiences, including so-called *ego dissolution experiences*, *unitive experiences,* and *mystical-type experiences*.

There is convergent evidence that high doses of psychedelic substances can elicit states of ego dissolution (Nour and Carhart-Harris, 2017; Millière et al., 2018). Ego dissolution is defined as a significant disruption of the sense of self (Nour and Carhart-Harris, 2017; Millière et al., 2018) to the point of temporary loss of one’s sense of self and self-world boundaries (Letheby and Gerrans, 2017; Millière, 2017, 2020). This experiential phenomenon is correlated with disintegration and desegregation on the neural systems level (Tagliazucchi et al., 2016; Carhart-Harris et al., 2016b). Related to this profound disruption of one’s sense of self, so-called “unitive experiences” signify a sense of personal, interpersonal, and existential interconnectedness (Nour and Carhart-Harris, 2017; Carhart-Harris et al., 2018). Interestingly, psilocybin administered to experienced meditators reliably induces this core element of the mystical-type experience (Smigielski et al., 2019a), which, in turn, is associated with changes in brain default-mode network connectivity and lasting behavioral effects (Smigielski et al., 2019b).

Mystical-type experiences, on the other hand, are rarely defined upfront but rather with relation to certain questionnaires targeting mystical experiences, such as the *Mystical Experience Questionnaire* (MEQ; Pahnke, 1969; MacLean et al., 2012; Barrett et al., 2015), the *Mysticism Scale* (Hood et al., 2001), and subscales of the *5-Dimension Altered States of Consciousness* questionnaire (*5D-ASC;* Dittrich, 1998). As an example, the MEQ comprises four subscales, inquiring into (1) a sense of unity or connectedness, (2) positive feelings such as love or peace, (3) alterations to the sense of time and space, and (4) ineffability, i.e., difficulty with articulating the experience with words (Barrett et al., 2015). In the field of psychedelic research, it has been repeatedly reported that psilocybin can occasion mystical-type experiences (Griffiths et al., 2006, 2008, 2011, 2016, 2018; Scheidegger, 2021a). However, the concept of the mystical experience has also become subject of debate in which potential risks from the scientific study of mystical experiences (Sanders and Zijlmans, 2021) are confronted with the objective of understanding the therapeutic effects of psychedelics and the tools available to study said effects (Breeksema and van Elk, 2021).

Notably, psychedelic studies, especially those with psilocybin, have repeatedly shown that participants frequently rate their psychedelic experiences as among the most meaningful in their lives (Griffiths et al., 2006, 2008, 2011, 2016, 2018; Ross et al., 2016). Scores on mystical-type experience questionnaires have been shown to predict treatment success at long-term follow-up in clinical studies (Garcia-Romeu et al., 2015; Griffiths et al., 2016; Davis et al., 2021; Ko et al., 2022). This underscores that the quality of subjective experience predicts positive mental health outcomes (Roseman et al., 2018) and may account for the majority of the lasting beneficial effects of psychedelics (Jungaberle et al., 2018; Yaden and Griffiths, 2021). These findings on the psychological and experiential level importantly suggest a potential *causal* role of the subjective experience on the therapeutic effects of psychedelics.

### 1.3. Connecting the various levels of psychedelic effects

These empirical findings on the biochemical, the neural, and the experiential level suggest that processes on each level may exhibit a causal role on the effects of psychedelics. This warrants the question how to relate these different levels of analysis to each other. van Elk and Yaden (2022) have recently distinguished two ways to relate these levels using two most common views in the current psychedelic literature: an *integration* view and a *pluralistic* view on causation.

*The integration* view on psychedelics targets a common pathway underlying the therapeutic effects of psychedelics for a variety of disorders. It aims to integrate various levels of description by converging them into a unified causal mechanism (van Elk and Yaden, 2022). One most influential integrative approach to psychedelic effects is the aforementioned REBUS model. The REBUS model describes a *cascade* of events, starting at the molecular level, then causing anatomical and functional changes in the brain that eventually culminate in the subjective experience of relaxed prior beliefs. In the words of Carhart-Harris (2019, p. 16), “it is proposed that psychedelics initiate a cascade of neurobiological changes that manifest at multiple scales and ultimately culminate in the relaxation of high-level beliefs”.

**Figure 1:**
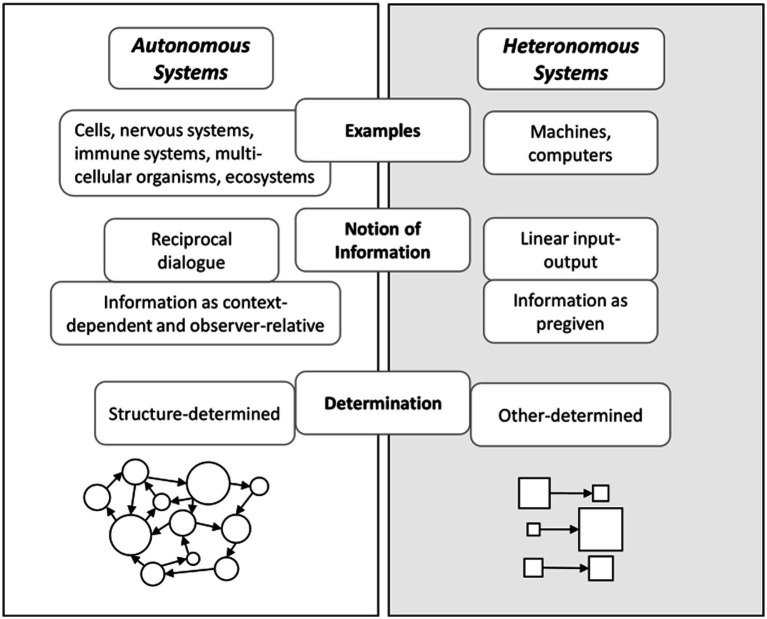
Autonomous systems in comparison to heteronomous systems.

Thereby, the REBUS model focuses on one specific aspect of the effects of psychedelics: the ability to acutely relax beliefs and assumptions. As a main cause of psychedelic effects, it suggests a single mechanism and reduces their complexity to a single cognitive process. While the REBUS model acknowledges the significance of how other factors should be considered for a comprehensive understanding of psychedelic effects, it proposes a specific neural mechanism as a key factor in shaping the therapeutic effects of psychedelics (see Figure 1). This single-pathway focus can be considered reductionist. When reduced to its proposition of a key neural mechanism leading to the relaxation of prior assumptions and expectations, the REBUS model implies a unidirectional linear molecule-to-brain and brain-to-experience relationship. The psychedelic molecule creates changes in brain activity and the brain creates the psychedelic experience.

The REBUS model, as one integrative view, has provided an influential and important contribution to the field of psychedelic research, incorporating significant insights from the entropic brain hypothesis, the free-energy principle, and predictive processing. Simultaneously, this integrative view has also received criticism. While the REBUS model illuminates a single neural mechanism, it may neglect other important aspects, other possible mechanisms, and the multi-faceted subjective nature of psychedelic experiences.

*The pluralistic* or *holistic* view on psychedelics shares this critique by emphasizing that psychedelic experiences cannot be reduced to a single cause-and-effect pathway: It needs an account of the interplay of multiple causal pathways to comprehensively understand a given phenomenon (Johnson et al., 2019). Accordingly, the pluralistic view, such as the biopsychosocial model to psychiatry, emphasizes the complex, multidimensional nature of causation involving multiple factors at different levels including biological, psychological, and social influences (Engel, 1977). Applied to psychedelic research, pluralistic theories of causation emphasize that social, cultural, and historical factors need to be considered in the study of psychedelic experiences (van Elk and Yaden, 2022). Likewise, it is argued that psychopharmacology needs to embrace interactions at various levels through multiple top-down and bottom-up causal pathways in order to account for therapeutic effects (Aftab and Stein, 2022).

While the integration view and the pluralistic view are distinct perspectives, they are not mutually exclusive. The causal mechanism described by the REBUS model to account for therapeutic effects of psychedelics can be seen as one of the multiple factors identified by the pluralistic view. The pluralistic view can incorporate the integration view but the integration view, if postulating a mono-causal cascade, cannot accommodate the pluralistic view.

Moreover, it has been argued that, in order to fully account for the multi-faceted nature of psychedelic experiences, a pluralistic theory of experience may be needed (van Elk and Yaden, 2022). In support of the pluralistic inclination toward the complexity and multidimensionality of causation, we wish to address this need through contributing a third and distinct but complementary and promising perspective on causality in psychedelic experiences: an enactive view. We argue that the enactive view on psychedelics complements the integration view by adding multiple pathways from the psychological and contextual level, while enriching the pluralistic view by explaining how biopsychosocial levels interact.

### 1.4. The enactive approach

The enactive approach is a cognitive science framework that has its origin in *The Embodied Mind* from 1991 by Varela, Thompson, and Rosch. Since then, it has gained substantial influence in the field of cognitive science. In this article, when referencing “the enactive approach,” we will refer to the work by Varela et al. (2017) and to its consistent advancement by Thompson (2007) and Di Paolo et al. (2010) among others. The enactive approach has its roots in various fields, including the autopoiesis theory by Maturana and Varela, phenomenological philosophy such as Merleau-Ponty’s *Phenomenology of Perception* and Hans Jonas’ philosophy of life. Moreover, it is inspired by dynamical systems theory and complex system science (Thompson, 2007) and Buddhist *Abhidharma* and *Mādhyamaka* philosophies (Varela et al., 2017). In recent years, the enactive approach has been applied to psychiatry (de Haan, 2020), placebo effects (Arandia and Di Paolo, 2021), pain (Stilwell and Harman, 2019; Coninx and Stilwell, 2021), consciousness research (Høffding and Martiny, 2016; Høffding et al., 2022; Valenzuela-Moguillansky and Demšar, 2022), artistic practices including dance and music improvisation (Schiavio and Høffding, 2015; Ravn and Høffding, 2022), meditation experiences (Meling, 2021, 2022), and ethics (Di Paolo and De Jaegher, 2022) among other fields.

The enactive approach provides a network of interrelated ideas including its central concepts of *autonomy*, *dynamic co-emergence*, *sense-making*, *groundlessness*, and *experience* (Thompson, 2007; Di Paolo et al., 2010; Varela et al., 2017). We propose that these are highly relevant for conceptualizing psychedelic drugs and their relation to psychedelic experiences. Some of them we will elaborate on in the main section of this article. The enactive approach’s differentiating factor is that it requires us to confront the lack of ultimate foundations through consistently following the thread of interdependence in various seemingly dualistic relationships, including those between parts and whole, self and world, and subject and object (Varela et al., 2017).

While the enactive approach has been widely ignored in the field of psychedelic research, the free-energy principle (FEP) and predictive processing (PP), two other recent and interrelated developments in cognitive science, have been very influential on neuroscientific research on psychedelics as they inspired the aforementioned REBUS model (Carhart-Harris et al., 2014; Carhart-Harris and Friston, 2019). Despite frequent claims from several authors about the compatibility between the FEP (or PP) and theories of autopoiesis and enaction (Clark, 2015; Allen and Friston, 2018; Constant et al., 2021; Korbak, 2021; Ramstead et al., 2021; Wiese and Friston, 2021), this has been recently criticized as misrepresenting enactive concepts (Di Paolo et al., 2022). While the details of this comparison go beyond the scope of this article, it underscores that applying FEP to psychedelics does *not* already entail applying the enactive approach to psychedelics. Therefore, a thorough application of enactive ideas to psychedelics is yet to be accomplished.

### 1.5. Aim and research questions

The main aim of this article is to explore psychedelics from an enactive perspective and more specifically to provide an enactive view on the causal relationship between psychedelic drugs, neural events, and experience. This aim is approached *via* the following two interconnected *research questions*:

1. What is the causal relationship between the psychedelic drug and brain activity?
2. What is the causal relationship between brain activity and the psychedelic experience?

## 2. An enactive approach to the psychedelic molecule-brain-experience relationship

In this section, we will address the two main research questions through analyzing the causal foundations of the psychedelic molecule-brain-experience relationships.

### 2.1. The molecule-brain relationship

In this subsection we address the first main research question. Guided by an enactive perspective, we re-evaluate the relationship between the psychedelic molecule and neural events through inquiring whether (1) the psychedelic molecule determines brain activity and (2) whether the psychedelic molecule transmits information.

#### 2.1.1. Does the molecule determine brain activity?

The *first question* of whether the psychedelic molecule determines brain activity can also be addressed through the enactive concept of *autonomy*.

*Autonomy* signifies how living cognitive systems are organized to generate and sustain themselves as an identity (Varela, 1997; Thompson and Stapleton, 2009). Autonomous systems are found at various levels of systems, including single cells, microbial communities, nervous systems, immune systems, multicellular organisms (such as humans), and ecosystems (Thompson, 2007). One way to define an autonomous system is with regard to its *operational closure*: An autonomous system is constituted by interacting processes that (1) recursively depend on each other for sustaining their network of activity, (2) constitute an identity or unity (e.g., a cell or neural pattern), and (3) determine the range of possible interactions with the environment (Thompson, 2007; Thompson and Stapleton, 2009).

**Figure 2:**
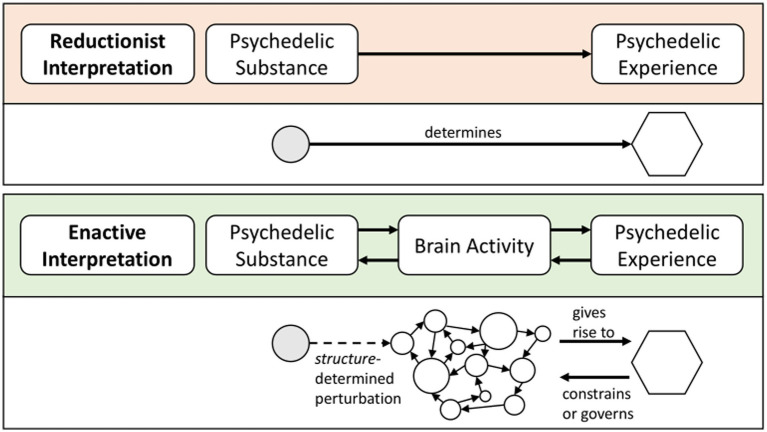
The psychedelic substance-experience link in a reductionist interpretation versus an enactive interpretation. In the enactive interpretation panel, links to the social and cultural environment are left out for conciseness purposes. However, the enactive approach includes an emphasis on the multiple interdependencies with the environment.

This brings us to the core of the distinction between autonomous systems and heteronomous systems (see Figure 2). A heteronomous system is determined from the outside whereas an autonomous system is not. An autonomous system is self-determining in its interactions with its environment (Thompson, 2007). Accordingly, the state of an autonomous system depends on how it *interacts* with perturbations from its environment.

This structural determination of an autonomous system is especially relevant for our discussion of the *psychedelic* molecule-brain relationship: An autonomous system is not determined from the outside. Its state depends on the specific organism-environment interactions. Fittingly for our discussion on psychedelics, Maturana and Varela (1987) illustrated this point with the example of a cell including a “*molecule X*” into its autonomous organization: The consequences of the cell including a molecule X into its processes are not merely determined by the specific features of the molecule X but by how the particular structure of the cell *interacts* with this molecule when integrating it. This example applies to every kind of autonomous system, including the nervous system integrating a psychedelic molecule.

Superimposed over the psychedelic molecule-brain relationship, this concept of autonomy has important implications: The consequences of the human nervous system integrating a psychedelic molecule into its autonomous activity is *not* determined by the specific features of the psychedelic substance but by the interaction between the nervous system’s particular autonomous activity and the psychedelic molecule. Therefore, the assumption of a linear causal impact of a psychedelic molecule on neural processes does not hold under the premise of autonomy. Autonomy confronts us with the lack of mono-causal foundation in the psychedelic molecule.

#### 2.1.2. Does the molecule transmit specific information to the brain?

The *second question* of whether the psychedelic molecule transmits *information* can be addressed through the enactive framing of information related to its concept of autonomy.

*Information* in autonomous systems is different from information in heteronomous systems. A heteronomous system (including computers) operates in an input–output manner. It takes information from an outside world, processes it, and generates output. In contrast, an autonomous system does not work in a linear input–output manner (Thompson, 2007). Figure 2 summarizes these differences between autonomous systems and heteronomous systems. In an autonomous system, perturbations are structure-determined. Therefore, it rather works in a dialogical manner: For autonomous systems, information does not belong to the environment. Rather, it belongs to the *system-environment coupling*. Importantly, what counts as information is determined by the way the autonomous system’s structure interacts with its environment. This includes the system’s history of becoming this temporary structure (Di Paolo, 2021), and its particular needs in its environment. Therefore, for an autonomous system information is context-dependent and observer-relative (Thompson, 2007, pp. 51–52).

This autonomy-informed notion of information has major implications for the psychedelic molecule-brain relationship. All living animals including humans are autonomous systems. In interaction with them, the psychedelic drug does not transmit information to the brain or body. Information does not belong to the psychedelic molecule. It must belong to the coupling of the psychedelic molecule with the structure of the autonomous system. Therefore, psychedelic drug effects are context-dependent and agent-relative.

### 2.2. The brain-experience relationship

In this subsection, we address the second main research question. From an enactive perspective, we re-evaluate the relationship between neural events and experience through inquiring whether the psychedelic-influenced brain determines the psychedelic experience. This can be addressed through the enactive concepts of *autonomy* and *dynamic co-emergence*.

#### 2.2.1. Does the brain determine experience?

A traditional neuroscientific approach to consciousness assumes a one-way causality from neural activity to consciousness. In contrast, the enactive approach proposes a two-way reciprocal relationship:

*“[w]e propose that there are two-way or reciprocal relationships between neural events and conscious activity. An attractive feature of this proposal is that it allows consciousness to be a causally efficacious participant in the cycles of operation constituting the agent’s life.”* (Thompson and Varela, 2001, p. 425).

The enactive approach arrives at this conclusion *via* applying the ideas of autonomy and dynamic co-emergence to neural assemblies and lived experience.

*Dynamic co-emergence* describes how an autonomous system works, signifying how an autonomous system as a *whole* is connected to the interaction of its *parts*. While there are different types and definitions of emergence, we focus here on a particular notion of emergence that emphasizes self-organization and circular causality and which is implicit in the concept of autonomy (Thompson, 2007). This notion of dynamic co-emergence involves a reciprocal influence of both a *local-to-global determination* (or “upward causation”) and a *global-to-local determination* (or “downward causation”) (Thompson, 2007). Local-to-global determination signifies that the parts give rise to the whole. This gives rise to novel processes that have “their own features, lifetimes, and domains of interaction” (Thompson and Varela, 2001, p. 419). Global-to-local determination signifies that the whole gives rise to the parts: Global characteristics of a system constrain or govern local interactions. Dynamic co-emergence refers to the idea that both local-to-global determination and global-to-local determination apply simultaneously (Thompson and Varela, 2001).

Importantly, the enactive approach proposes that this dynamic co-emergence applies to the brain-experience relationship:

*[g]iven that the coupled dynamics of brain, body and environment exhibit self-organization and emergent processes at multiple levels, and that emergence involves both upward and downward causation, it seems legitimate to conjecture that downward causation occurs at multiple levels in these systems, including that of conscious cognitive acts in relation to local neural activity. Indeed, this point has been noted by authors concerned with the dynamical systems approach to cognition.* (Thompson and Varela, 2001, p. 421, emphasis added).

This dynamic co-emergence between local neural activity and global conscious cognitive acts implies that lived experience co-emerges with brain activity while being irreducible to it. Experience is not an epiphenomenal effect of neural activity but rather has causal efficacy. As an example for downward causation, Thompson and Varela (2001) present an experimental case study showing a particular way how experiential acts of perception can pull epileptic activities toward unstable periodic orbits. As a co-emergent phenomenon, experience gains its own characteristics and causal global-to-local efficacy on constraining local neural activity: Neural activity and lived experience co-emerge.

Applied to the psychedelic brain-experience relationship, the dynamic co-emergence between neural events and consciousness provides obvious implications: The psychedelic lived subjective experience is *not* epiphenomenal. It is not linearly determined by the neural activity in reaction to a psychedelic drug. Taking the concept of dynamic co-emergence seriously and applying it to the case of psychedelic experiences implies a *reciprocal causal relationship* between neural events and the psychedelic experience: While neural events (in structure-determined interaction with a psychedelic drug) give rise to the psychedelic experience as experiential cognitive acts (*via* local-to-global determination), these psychedelic experiential cognitive acts simultaneously limit the possible neural activity (*via* global-to-local determination). Therefore, the assumption that *the brain affects experience* through a one-way causal relationship does not hold under the premise of dynamic co-emergence. Psychedelic neural activity and psychedelic experiences co-emerge and dialogically affect each other.

#### 2.2.2. Circular causality in the psychedelic brain-experience relationship

This notion of circular causality underlying the concept of dynamic co-emergence is well expressed in Merleau-Ponty’s (1963, p. 50) *Structure of Behavior*: “The genesis of the whole by compositions of the parts is fictitious. It arbitrarily breaks the chain of reciprocal determinations”. He distinguishes between linear causality and circular causality. Applied to the psychedelic brain-experience relationship, *linear causality* would imply that neural activity determines the psychedelic experience, but the psychedelic experience does *not* determine neural activity. In contrast, *circular causality* implies that neural activity and the psychedelic experience are part of the same system and therefore determine each other mutually. In psychology and biology, causality is never linear or mechanistic but circular (Merleau-Ponty, 1963; Thompson, 2007). This circular causal notion applies to both the psychedelic molecule-brain relationship and the psychedelic brain-experience relationship.

This view of circular causality is echoed in the *interventionist approach* to causation: If X is a cause of Y it must apply that by intervening on X we also intervene on Y. This interventionist approach can be applied to the brain-experience relationship (Thompson, 2009). First, intervening on biological events has consequences on one’s experience. Triggering a change in one’s *biological* state (for example by a psychedelic drug) may result in short-term or long-term changes in one’s *experiential* state. Second, intervening on one’s experience has consequences on one’s biological state. Triggering a change in one’s *experiential* state by purely psychological means (for example by contemplative training or psychotherapy) may result in short-term or long-term changes to neural activity or hormonal patterns. In other words, to intervene on X is to intervene on Y; to intervene on Y is to intervene on X. They are part of the same system. Therefore, circular causality applies.

### 2.3. Summary on the specific research questions

In exploring the first research question, we applied the concept of *autonomy* to the *psychedelic molecule-brain relationship* (research question 1): The consequences of the human nervous system integrating a psychedelic molecule into its autonomous activity is *not* mono-causally determined by the specific features of the psychedelic substance but by the particular interaction between the temporary structure of the nervous system’s autonomous activity and the psychedelic molecule. The consequences neither belong to the psychedelic molecule nor to the nervous system’s activity. They depend on the specific molecule-organism interaction.

In exploring the second research question, we applied the concept of *dynamic co-emergence* to the *psychedelic brain-experience relationship* (research question 2): The psychedelic experience is not epiphenomenal or linearly determined by the neural activity in reaction to a psychedelic drug. Rather, they are co-dependently emerging. Neural events give rise to the psychedelic experience and the psychedelic experience limits the range of potential neural events. Rather than being a mere epiphenomenon of neural activity, the psychedelic experience is causally efficacious on neural activity: Neural activity and experience are co-emergent. As with the psychedelic molecule-brain relationship, from an enactive perspective the psychedelic brain-experience relationship is not unidirectional but bidirectional and circular (see Figure 1).

The inquiry into both research questions from an enactive view resulted in a perspective that emphasizes interdependence. Therefore, the main contribution of this article is a framework of ideas that accounts for the interdependence of psychedelic experiences including their nonlinear causality and non-reducibility. This results in a complex account of causality in psychedelic experiences.

## 3. Implications for psychedelic research and psychedelic therapy

The points presented here used the enactive view to offer an alternative perspective on causality in the psychedelic molecule-brain-experience relationship giving rise to important implications for the field of psychedelic research and for an understanding of therapeutic effects of psychedelics.

The presented enactive approach to psychedelics contributes a valuable perspective to psychedelic research in multiple ways. First, the enactive view complements the integration view as it accounts for empirical findings that cannot be explained by the REBUS model alone or by integrative or reductionist approaches in general. Second, it enriches pluralistic theories of causation by explaining how exactly processes on different levels (biological, psychological, and social processes) can causally interact. Third, the enactive view on psychedelics accounts for a plurality of causes of therapeutic effects in psychedelic-assisted therapy and thereby embraces a plurality of treatment forms. Fourth, from this perspective, suggestions for future research can be derived including further research questions.

### 3.1. The enactive approach and the integration view

While the REBUS model, as one exemplary integration view, proposes that the biochemical, neural, and experiential aspects of psychedelics converge on a single causal cascade, there is also contradicting evidence. First, while there are studies corroborating the REBUS model through showing LSD-induced reductions in electrophysiological responses to surprising stimuli (Timmermann et al., 2018), other studies have not observed such reductions in surprise responses (Umbricht et al., 2003; Vollenweider and Preller, 2020). Second, while the REBUS model proposes that effects on the default-mode network (DMN) are central in psychedelics, even stronger effects have been reported in changing activity in other networks (Lebedev et al., 2016; Mason et al., 2020). Third, psychedelics may elicit a broad variety of experiences rather than only the relaxation of high-level beliefs. It has been argued that psychedelics, depending on dosage, may also lead to a strengthening of beliefs (Safron, 2020). Fourth, there is evidence suggesting a potential causal role of subjective experiences on therapeutic outcomes (Roseman et al., 2018; Yaden and Griffiths, 2021). The REBUS model, however, with its focus on a key neural mechanism does not account for a causal role of the quality of subjective experience. Fifth, the role of *set and setting*, i.e., extrapharmacological or non-biological factors that shape the response to psychedelics, are increasingly recognized in psychedelic research: The expectation, preparation, and intention (set) and the physical and social environment (setting) affect to some degree the consequences of the psychedelic substance-human interaction (Hartogsohn, 2016, 2017). This relevance of set and setting is not captured in an integration account that focuses on a single causal neural pathway.

These findings suggest other causal pathways, creating tension with the integrative proposal that the different levels converge on a single causal pathway. From an enactive perspective, however, these empirical findings confirm the causal complexity that mediates psychedelic effects: As an example, the aforementioned measured surprise response depends not only on the substance but also on the instruction of the task, on the participants’ expectations and intentions, on their felt relationship to the researchers, their individual bodily constitution, their history of interactions with their environment, and many more factors. Keeping this complexity of causal influences in mind, it is not surprising that some studies showed psychedelic-induced reductions in electrophysiological responses to surprising stimuli (Timmermann et al., 2018), while other studies have not observed such reductions in surprise responses (Umbricht et al., 2003; Vollenweider and Preller, 2020). The same applies to the mentioned finding of a variety of brain networks that show changed activity and to the finding that psychedelics may not only lead to a relaxation of beliefs but also to their strengthening. Depending on other causal influences, as from the body, the environment, the experience, the measured effects naturally vary. And the enactive approach can accommodate this variety of causal influences as its emphasis on the dynamic co-emergence between different levels suggests bidirectional causality on multiple levels. First, neural activity is not dependent only on the substance but on the entire body’s constitution, other perturbations from the environment, the subject’s lived experience, and many further factors that cannot be entirely controlled within an empirical study. Neural activity under the effects of a psychedelic substance is contextual and situated: Therefore, it strongly varies across studies. Second, from an enactive perspective subjective experiences co-emerge with neural processes so that neural processes have causal efficacy on subjective experience while subjective experience has causal efficacy on neural processes. Third, the enactive account emphasizes the organism-environment interaction that shapes cognition. This includes a causal role of extrapharmacological factors, such as psychological, social, and historical factors that shape the psychedelic experience.

From an enactive view, the integrative view that “psychedelics initiate a cascade of neurobiological changes that manifest at multiple scales and ultimately culminate in the relaxation of high-level beliefs” (Carhart-Harris, 2019, p. 16) is not negated.^1^ Rather, it is expanded through adding multiple causal pathways in order to capture the causal complexity that contributes to a psychedelic experience. This focus on the complexity and multidimensionality of causation is shared between the pluralistic view and the enactive approach.

### 3.2. The enactive approach and the pluralistic view

The pluralistic view and the enactive approach have several commonalities regarding the notion of causality. Both approaches emphasize causal complexity and that multiple factors contribute to psychedelic effects, including the environmental setting, the socio-cultural context, and the individual’s expectations. In other words, both approaches consider the context-dependency and observer-relativity of a psychedelic experience and acknowledge the importance of the subjective experience.

However, as argued by de Haan (2021) in the context of psychiatry, the biopsychosocial model as one pluralistic view of causation faces an *integration problem,* as it does not tell how processes of largely differentiating natures can causally affect each other. The enactive approach, however, can explain such biopsychosocial interaction (de Haan, 2020). Likewise, the enactive approach substantially enriches the pluralistic view of causation regarding psychedelics as it provides a principled account of how biochemical, neural, and experiential processes affect each other through local-to-global and global-to-local determination. They are mutually dependent autonomous processes that co-emerge and therefore exert circular causality. These interactions on multiple levels shape the psychedelic experience.

### 3.3. Psychedelic-assisted psychotherapy from an enactive perspective

In acknowledging the plurality of causes on therapeutic effects, the enactive view provides a theoretical basis for a holistic approach to psychiatry (de Haan, 2020). Rather than advocating a specific form of treatment, the enactive view of therapy targets a better understanding of how various influences interact. Accordingly, it embraces a plurality of treatment forms in a personalized approach that targets the dynamic and complex person-world system as the unit of analysis (de Haan, 2020).

Likewise, the presented enactive approach to causality in the psychedelic molecule-brain-experience relationship suggests similar implications for psychedelic therapy. In acknowledging the multiple and circular causal pathways between the psychedelic substance, neural activity, the subjective experience, the social and physical environment, and many other factors, the enactive approach embraces a plurality of ways of how therapeutic effects can emerge in psychedelic-assisted psychotherapy. In principle, it can be derived that therapeutic effects can co-emerge with a variety of causal factors and their mutual interactions, including a particular molecular substance effect (e.g., binding on 5-HT_2A_ receptors), a particular pattern of neural activity (e.g., activity in the claustro-cortical circuit), particular experiences (e.g., a mystical-type experience), a particular bodily state (e.g., a comfortable and relaxed body posture or a release of muscular tension), a particular physical environment (e.g., a calm and warm space with soothing music), a particular social context (e.g., a friendly, accepting, and nonjudgmental atmosphere), and a particular intention or expectation (e.g., the intention to turn one’s attention to unpleasant aspects of one’s life in an accepting and nonjudgmental way). Therefore, the enactive view supports a holistic approach to psychedelic-assisted psychotherapy that acknowledges the potentially therapeutic effects of psychedelic substances and simultaneously goes beyond the focus on the psychedelic substance through emphasizing the importance of the ecology of causes that surrounds the organism’s interaction with the psychedelic substance.

### 3.4. Recommendations for future research

Finally, suggestions for a psychedelic research agenda can be derived from the enactive view on the psychedelic molecule-brain-experience relationship. As we have seen, the enactive view admits that there is definitely an impact on experience from the molecule *via* neural activity. Simultaneously, it prompts us to acknowledge the vast complexity of causes and conditions that enable a trial participant to report a certain psychedelic experience or therapeutic improvements. The enactivist insistence on the complexity of causes that shape a psychedelic experience prompts questions that go beyond the reduction to the drug or to the brain: How did the neural system take up this molecule, what was the structure of the nervous system integrating that psychedelic substance? What impacted that structure of the nervous system, e.g., other bodily processes or environmental factors? Under which conditions were these experiences reported? What were the expectations of the participants, what was their environmental context?

Further research is needed to operationalize these questions and other implicit hypotheses from this article in order to empirically test them; employing a compatible scientific methodology that connects different views and disciplinary methodologies in a way that acknowledges the enactive view of circular causality.

Accordingly, psychedelic research inspired by the enactive approach may require an interdisciplinary endeavor that integrates different perspectives, such as neuroscientific, phenomenological, cultural, anthropological, and historical, among others, in order to provide a more comprehensive understanding of the complex interactions across different levels involved in the psychedelic experience.

## 4. Conclusion

The enactive view on psychedelics complements the integration view by adding multiple pathways from the psychological and contextual level, while enriching the pluralistic view by explaining how biopsychosocial levels interact. Through its concepts of autonomy and dynamic co-emergence, the enactive approach offers the possibility for a circular causality between multiple levels, including a causal efficacy of subjective experience on neural activity. As a holistic approach, the enactive perspective targets an understanding of how various influences interact in therapeutic practice and thereby embraces a plurality of ways that can support therapeutic effects in psychedelic therapy. Finally, from an enactive view, an interdisciplinary integration of various perspectives and methods on various levels may be required for future psychedelic research in order to address the complex causal circularity involved in the psychedelic molecule-brain-experience relationship. Insights into a variety of causes at play in the psychedelic substance-organism interaction are a critical step toward fully harnessing the therapeutic potential of psychedelics.

## Data availability statement

The original contributions presented in the study are included in the article, further inquiries can be directed to the corresponding author.

## Author contributions

DM conceptualized and wrote the first and subsequent drafts of the manuscript. MS contributed to manuscript revision and wrote sections of the manuscript. All authors contributed to the article and approved the submitted version.

## Funding

This work is supported by a Spark Grant from the Swiss National Science Foundation (CRSK-1_196833), by the BIAL Foundation (no. 333/20), and by the Reconnect Foundation.

## Conflict of interest

MS declares that he co-founded Reconnect Labs, an academic spin-off at the University of Zurich, focused on the development of psychedelic medicines for mental health. DM declares that this research was conducted in the absence of any commercial or financial relationships that could be construed as a potential conflict of interest.

The handling editor NL declares a past co-authorship with the author MS.

## Publisher’s note

All claims expressed in this article are solely those of the authors and do not necessarily represent those of their affiliated organizations, or those of the publisher, the editors and the reviewers. Any product that may be evaluated in this article, or claim that may be made by its manufacturer, is not guaranteed or endorsed by the publisher.

## Footnotes

^1^We are aware that many enactivist thinkers consider their position as a paradigm shift that ought to replace a representationalist view of mind and brain (*cf.* Thompson, 2007; Di Paolo et al., 2017; Gallagher, 2017). While the representationalist view is rejected (as it reduces the causal complexity of the organism-environment interaction that is involved in perception to a unidirectional and passive environment-to-organism imprint), the enactive approach generally acknowledges a (relevant but insufficient) role of environmental perturbations on the organism’s perception. Therefore, we speak of "complementing" the integration view through considering multiple pathways from the psychological and contextual level. This implies, however, a rejection of incorporating the integration view into a reductionist framework. While it is not within the scope of this paper to discuss the nuances between different forms and interpretations of the enactive approach (see De Jesus, 2016) or even of 4E cognition and their varied stances on the explanatory need for representations, we simply note that the integration view is explanatorily insufficient to accommodate all the aforementioned experimental results and that other causal mechanisms, including those advanced by the enactive approach, must be invoked to explain these results.
